# Orthogonal Stochastic Duality Functions from Lie Algebra Representations

**DOI:** 10.1007/s10955-018-2178-7

**Published:** 2018-10-19

**Authors:** Wolter Groenevelt

**Affiliations:** Technische Universiteit Delft, DIAM, PO Box 5031, 2600 GA Delft, The Netherlands

**Keywords:** Stochastic duality, Lie algebra representations, Hypergeometric functions, Orthogonal polynomials

## Abstract

We obtain stochastic duality functions for specific Markov processes using representation theory of Lie algebras. The duality functions come from the kernel of a unitary intertwiner between $*$-representations, which provides (generalized) orthogonality relations for the duality functions. In particular, we consider representations of the Heisenberg algebra and $\mathfrak {su}(1,1)$. Both cases lead to orthogonal (self-)duality functions in terms of hypergeometric functions for specific interacting particle processes and interacting diffusion processes.

## Introduction

A very useful tool in the study of stochastic Markov processes is duality, where information about a specific process can be obtained from another, dual, process. The concept of duality was introduced in the context of interacting particle systems in [17], and was later on developed in [15]. For more applications of duality see e.g. [4, 6, 13, 18].

Two processes are in duality if there exists a duality function, i.e. a function of both processes such that the expectations with respect to the original process is related to the expectations with respect to the dual process (see Sect. 2 for a precise statement). Recently in [5, 16] orthogonal polynomials of hypergeometric type were obtained as duality functions for several families of stochastic processes, where the orthogonality is with respect to the corresponding stationary measures. These orthogonal polynomials contain the well-known simpler duality functions (in the terminology of [16], the classical and cheap duality functions) as limit cases. In [5], Franceschini and Giardinà use explicit relations between orthogonal polynomials of different degrees, such as raising and lowering formulas, to prove the stochastic duality. In [16], Redig and Sau find the orthogonal polynomials using generating functions. With a similar method they also obtain Bessel functions, which are not polynomials, as self-duality function for a continuous process.

The goal of this paper is to demonstrate an alternative method to obtain the orthogonal polynomials (and other ‘orthogonal’ functions) from [5, 16] as duality functions. The method we use is based on representation theory of Lie algebras. This is inspired by [3, 7], where representation theory of $\mathfrak {sl}(2,{\mathbb {C}})$ and the Heisenberg algebra is used to find (non-orthogonal) duality functions, see also Sturm et al. [19] for a Lie algebraic approach to duality. Roughly speaking, the main idea is to consider a specific element *Y* in the Lie algebra (or better, enveloping algebra). Realized in two different, but equivalent, representations $\rho$ and $\sigma$, $\rho (Y)$ and $\sigma (Y)$ are the generators of two stochastic processes. In case of $\mathfrak {sl}(2,{\mathbb {C}})$, *Y* is closely related to the Casimir operator. The duality functions come from an intertwiner between the two representations. In this paper we consider a similar construction with unitary intertwiners between $*$-representations, so that the duality functions will satisfy (generalized) orthogonality relations.

In Sect. 2 the general method to find duality functions from unitary intertwiners is described. In Sect. 3 the Heisenberg algebra is used to show duality and self-duality for the independent random walker process and a Markovian diffusion process. The self-duality of the diffusion process seems to be new. The (self-)duality functions are Charlier polynomials, Hermite polynomials and exponential functions. In Sect. 4 we consider discrete series representation of $\mathfrak {su}(1,1)$, and obtain Meixner polynomials, Laguerre polynomials and Bessel functions as (self)-duality functions for the symmetric inclusion process and the Brownian energy process. We would like to point out that the self-duality functions are essentially the (generalized) matrix elements for a change of base between bases on which elliptic or parabolic Lie group / algebra elements act diagonally, see e.g. [2, 14], so in these cases stochastic self-duality is a consequence of a change of bases in the representation space.

### Notations and Conventions

By ${\mathbb {N}}$ we denote the set of nonnegative integers. We often write *f*(*x*) for a function $x\mapsto f(x)$; the distinction between the function and its values should be clear from the context. For functions $x\mapsto f(x;p)$ depending on one or more parameters *p*, we often omit the parameters in the notation. For a set *E*, we denote by *F*(*E*) the vector space of complex-valued functions on *E*. ${\mathcal {P}}$ is the vector space consisting of polynomials in one variable.

We use standard notations for shifted factorials and hypergeometric functions as in e.g. [1]: for $a \in {\mathbb {C}}$ and $n \in {\mathbb {N}}$ the shifted factorial $(a)_n$ is defined by

$$\begin{aligned} (a)_0=1,\qquad (a)_n= a (a+1) \cdots (a+n-1), \end{aligned}$$

and the hypergeometric series ${}_rF_s$ is defined by

$$\begin{aligned} \,_{r}F_{s}\!\left( \genfrac{}{}{0.0pt}{}{a_1,a_2,\ldots ,a_r}{b_1,b_2\ldots ,b_s} \,;x \right) = \sum _{n=0}^\infty \frac{ (a_1)_n (a_2)_n \cdots (a_r)_n }{(b_1)_n (b_2)_n \cdots (b_s)_n } \frac{ x^n }{n!}. \end{aligned}$$

See e.g. [1, Section 2.1] for convergence properties of this series. Note that if $a_i \in -{\mathbb {N}}$ for some *i*, then the series is a finite sum.

## Stochastic Duality Functions from Lie Algebra Representations

In this section we describe the method to obtain stochastic duality functions from $*$-representations of a Lie algebra. This method will be applied in explicit examples in Sects. 3 and 4 .

### Stochastic Duality

Let $X_1=\{\eta _1(t) \mid t>0\}$ and $X_2= \{ \eta _2(t) \mid t>0\}$ be stochastic Markov processes with state spaces $\Omega _1$ and $\Omega _2$, respectively. These processes are in duality if there exists a duality function $D:\Omega _1\times \Omega _2 \rightarrow {\mathbb {C}}$ such that for all $t>0$, $\eta _1$ and $\eta _2$, the relation

$$\begin{aligned} {\mathbb {E}}_{\eta _1}\big [ D(\eta _1(t),\eta _2) \big ] = {\mathbb {E}}_{\eta _2}\big [ D(\eta _1,\eta _2(t)) \big ] \end{aligned}$$

holds, where ${\mathbb {E}}_\eta$ represents the expectation. If $X_1=X_2$, the process is called self-dual. Let $L_1$ and $L_2$ be the infinitesimal generators of the two processes. Under appropriate conditions, see e.g. [10, Proposition 1.2], duality of the processes is equivalent to duality of the generators, i.e. 

$$\begin{aligned} {[L_1 D(\cdot ,\eta _2)]}(\eta _1) = [L_2 D(\eta _1,\cdot )](\eta _2), \qquad (\eta _1,\eta _2) \in \Omega _1 \times \Omega _2. \end{aligned}$$

If $L_1=L_2$, then the operator is self-dual.

In this paper, we consider processes with state space $\Omega = E_1 \times \cdots \times E_N$, where each $E_j$ is a subset of ${\mathbb {R}}$. Furthermore, the generators will be of the form

2.1 $$\begin{aligned} L = \sum _{i<j} L_{i,j} \end{aligned}$$

where $L_{i,j}$ is an operator on $F(E_i \times E_{j})$. This allows us to only consider operators acting on functions in two variables.

### Lie Algebra Representations

Let ${\mathfrak {g}}$ be a finite dimensional complex Lie algebra, with basis elements $X_1,\ldots ,X_n$ satisfying the commutation relations

$$\begin{aligned} {[X_i,X_j]}=\sum _{k=1}^n c_{ijk} X_k,\qquad 1 \le i < j \le n, \end{aligned}$$

for certain coefficients $c_{ijk} \in {\mathbb {C}}$. The universal enveloping algebra $U({\mathfrak {g}})$ is the associative algebra with unit element generated by $X_1,\ldots , X_n$ subject to the relations

$$\begin{aligned} X_i X_j - X_j X_i = \sum _{k=1}^n c_{ijk} X_k,\qquad 1 \le i < j \le n. \end{aligned}$$

We assume ${\mathfrak {g}}$ has a $*$-structure, i.e. there exists an involution $X \mapsto X^*$ such that

$$\begin{aligned} (aX+bY)^* = {\overline{a}}X^* + {\overline{b}}Y^*, \qquad [X,Y]^* =[Y^*,X^*], \qquad X,Y \in {\mathfrak {g}},\ a,b \in {\mathbb {C}}. \end{aligned}$$

The $*$-structure of ${\mathfrak {g}}$ extends uniquely to a $*$-structure of $U({\mathfrak {g}})$, i.e.

$$\begin{aligned} (aX+bY)^* = {\overline{a}}X^* + {\overline{b}}Y^*, \qquad (XY)^* =Y^*X^*, \qquad X,Y \in U({\mathfrak {g}}),\ a,b \in {\mathbb {C}}. \end{aligned}$$

Let $\rho$ be a representation of ${\mathfrak {g}}$ on the vector space *F*(*E*). We call $\rho$ a $*$-representation of ${\mathfrak {g}}$ on ${\mathcal {H}}=L^2(E,\mu )$ with dense domain ${\mathcal {D}} \subseteq {\mathcal {H}}$, if $\rho (X)$ is defined on ${\mathcal {D}}$ and $\langle \rho (X)f,g\rangle = \langle f, \rho (X^*) g \rangle$ for all $X \in {\mathfrak {g}}$ and all $f,g \in {\mathcal {D}}$. A $*$-representation of ${\mathfrak {g}}$ extends uniquely to a $*$-representation of $U({\mathfrak {g}})$ on ${\mathcal {H}}$ (with the same domain ${\mathcal {D}}$).

If $\rho _1$ and $\rho _2$ are $*$-representations of ${\mathfrak {g}}$ on ${\mathcal {H}}_1$ and ${\mathcal {H}}_2$ respectively, then $\rho$ defined by

$$\begin{aligned} \rho (X) = (\rho _1 \otimes \rho _2) (\Delta (X)), \qquad \Delta (X) = 1 \otimes X + X \otimes 1 \in U({\mathfrak {g}})^{\otimes 2}, \quad X \in {\mathfrak {g}}, \end{aligned}$$

is a $*$-representation of ${\mathfrak {g}}$ on ${\mathcal {H}}_1 \otimes {\mathcal {H}}_2$ (the Hilbert space completion of the algebraic tensor product of ${\mathcal {H}}_1$ and ${\mathcal {H}}_2$). Furthermore, $\rho$ can be considered as a representation of $U({\mathfrak {g}})^{\otimes 2}$ by defining (slightly abusing notation)

$$\begin{aligned} \rho (X) = (\rho _1 \otimes \rho _2)(X), \qquad X \in U({\mathfrak {g}})^{\otimes 2}. \end{aligned}$$

We will often use the notation $\rho =\rho _1\otimes \rho _2$.

Two $*$-representations $\rho _1$ and $\rho _2$ are unitarily equivalent if there exists a unitary operator $\Lambda :{\mathcal {H}}_1 \rightarrow {\mathcal {H}}_2$ such that $\Lambda ({\mathcal {D}}_1) = {\mathcal {D}}_2$ and $\Lambda [\rho _1(X) f] = \rho _2(X) \Lambda (f)$ for all $X \in {\mathfrak {g}}$ and $f \in {\mathcal {D}}_1$.

#### Lemma 2.1

For $j=1,2$, let $\rho _j$ be representations of ${\mathfrak {g}}$ on $F(E_j)$ and $*$-representations of ${\mathfrak {g}}$ on $L^2(E_j,\mu _j)$ with domain ${\mathcal {D}}_j$. Suppose $K:E_1 \times E_2 \rightarrow {\mathbb {C}}$ is a function with the following properties:

1. $[\rho _1(X^*)K(\cdot ,y)](x) = [\rho _2(X) K(x,\cdot )](y)$ for all $X \in {\mathfrak {g}}$ and $(x,y) \in E_1 \times E_2$.
2. The operator $\Lambda :{\mathcal {D}}_1 \rightarrow L^2(E_2,\mu _2)$ defined by
  $$\begin{aligned} \Lambda f = \left( y \mapsto \int _{E_1} f(x) K(x,y)\, d\mu _1(x) \right) , \end{aligned}$$
  extends to a unitary operator $\Lambda : L^2(E_1,\mu _1) \rightarrow L^2(E_2,\mu _2)$.

Then $\rho _1$ and $\rho _2$ are unitarily equivalent $*$-representations of ${\mathfrak {g}}$ with intertwiner $\Lambda$.

#### Proof

This follows directly from

$$\begin{aligned} (\Lambda [\rho _1(X)f])(y) = \int _{E_1} [\rho _1(X)f](x) K(x,y) \,d\mu _1(x) = \int _{E_1} f(x) [\rho _1(X^*)K(\cdot ,y)](x) \, d\mu _1(x), \end{aligned}$$

and

$$\begin{aligned} {[\rho _2(X) (\Lambda f)]}(y) = \int _{E_1} f(x) [\rho _2(X)K(x,\cdot )](y) \, d\mu _1(x), \end{aligned}$$

using property 1. $$\square $$

### Duality from $*$-Representations

We are now ready to obtain duality functions for certain operators from the kernels of intertwining operators between $*$-representations.

#### Theorem 2.2

For $j\in \{1,\ldots ,N\}$ let $\rho _j$ and $\sigma _j$ be unitarily equivalent $*$-representations of ${\mathfrak {g}}$ on $L^2(E_j,\mu _j)$ and $L^2(F_j,\nu _j)$, respectively, such that the corresponding unitary intertwiner $\Lambda _j:L^2(E_j,\mu _j) \rightarrow L^2(F_j,\nu _j)$ is an integral operator as in Lemma 2.1, i.e.

$$\begin{aligned} (\Lambda _jf)(y) = \int _{E_j} f(x) K_j(x,y) \,d\mu _j(x) , \qquad \text {for } \nu _j-\text {almost all } y \in F_j, \end{aligned}$$

for some kernel $K_j:E_j\times F_j \rightarrow {\mathbb {C}}$ satisfying

2.2 $$\begin{aligned} {[\rho _j(X^*)K_j(\cdot ,y)]}(x)= [\sigma _j(X)K_j(x,\cdot )](y), \qquad (x,y) \in E_j \times F_j, \qquad X \in {\mathfrak {g}}. \end{aligned}$$

Furthermore, let

$$\begin{aligned} \Omega _1=E_1\times \cdots \times E_N, \qquad \Omega _2= F_1 \times \cdots \times F_N, \end{aligned}$$

and let $\mu$ and $\nu$ be the product measures on $\Omega _1$ and $\Omega _2$ given by

$$\begin{aligned} \mu =\mu _1 \times \cdots \times \mu _N, \qquad \nu = \nu _1 \times \cdots \times \nu _N, \end{aligned}$$

then $\rho =\rho _1 \otimes \cdots \otimes \rho _N$ and $\sigma =\sigma _1 \otimes \cdots \otimes \sigma _N$ are $*$-representations of $U({\mathfrak {g}})^{\otimes N}$ on $L^2(\Omega _1,\mu )$ and $L^2(\Omega _2,\nu )$, respectively. For $Y \in U({\mathfrak {g}})^{\otimes N}$, let $L_1$ and $L_2$ be the operators given by

$$\begin{aligned} L_1=\rho (Y^*), \qquad L_2=\sigma (Y). \end{aligned}$$

Then $L_1$ and $L_2$ are in duality, with duality function given by

$$\begin{aligned} D(x,y)=\prod _{j=1}^N K_j(x_j,y_j), \qquad x=(x_1,\ldots ,x_N) \in \Omega _1, \ y=(y_1,\ldots ,y_N) \in \Omega _2. \end{aligned}$$

#### Proof

Write $Y = \sum Y_{(1)} \otimes \cdots \otimes Y_{(N)}$, with $Y_{(j)} \in U({\mathfrak {g}})$. It is enough to verify that

$$\begin{aligned} {[\rho _j(Y^*_{(j)}) K_j(\cdot ,y_j)]}(x_j) = [\sigma _j(Y_{(j)}) K_j(x_j,\cdot )](y_j), \qquad (x_j,y_j) \in E_j \times F_j, \end{aligned}$$

for $j=1,2$. Since we have $Y_{(j)}= Y_{j,1}Y_{j,2} \cdots Y_{j,k_j}$ for certain $Y_{j,i} \in {\mathfrak {g}}$, the result follows from (2.2). $\square$

#### Remark 2.3

If the set $\Omega _1$ in Theorem 2.2 is countable, then the set of duality functions $\{D(x,\,\cdot \,\,)\mid x \in \Omega _1\}$ is an orthogonal basis of $L^2(\Omega _2,\nu )$. Indeed, write

$$\begin{aligned} \int _{\Omega _1} f(x) d\mu (x) = \sum _{x \in \Omega _1} w(x) f(x), \end{aligned}$$

and define $d_x(y)= \delta _{x,y}/w(x)$ for $x,y \in \Omega _1$, then $\{ d_x \mid x \in \Omega _1\}$ is an orthogonal basis for $L^2(\Omega _1,\mu )$ with squared norm $w(x)^{-1}$. Then by unitarity of $\Lambda = \Lambda _1 \otimes \ldots \otimes \Lambda _N$ and $(\Lambda d_x)(y) = D(x,y)$ it follows that $\{D(x,\cdot ) \mid x \in \Omega _1\}$ is an orthogonal basis for $L^2(\Omega _2,\nu )$.

In the following sections we apply Theorem 2.2 using explicit representations in terms of difference operators or differential operators. $L_1$ and $L_2$ will be generators of specific processes, and $\mu$ and $\nu$ are corresponding stationary measures. In particular, $L_1$ and $L_2$ are self-adjoint operator on $L^2(\Omega _1,\mu )$ and $L^2(\Omega _2,\nu )$ respectively. The main problem is finding explicitly the appropriate intertwiner. The algebra element *Y*, which is self-adjoint in $U({\mathfrak {g}})^{\otimes N}$, will always have a specific form corresponding to (2.1);

2.3 $$\begin{aligned} Y = \sum _{i<j} p_{i,j} {\hat{Y}}_{i,j}, \end{aligned}$$

with ${\hat{Y}}$ a self-adjoint element in $U({\mathfrak {g}})^{\otimes 2}$ and $p_{i,j}\ge 0$. Here we use leg-numbering notation: for $X=\sum X_{(1)} \otimes X_{(2)} \in U({\mathfrak {g}})^{\otimes 2}$ we denote by $X_{i,j} \in U({\mathfrak {g}})^{\otimes N}$ the element

$$\begin{aligned} X_{i,j} = \sum 1 \otimes \cdots \otimes 1 \otimes X_{(1)} \otimes 1 \otimes \cdots \otimes 1 \otimes X_{(2)} \otimes 1 \cdots \otimes 1, \end{aligned}$$

with $X_{(1)}$ in the *i*th factor and $X_{(2)}$ in the *j*th factor. In fact, we obtain duality between the operators $\rho ({\hat{Y}}_{i,j})$ and $\sigma ({\hat{Y}}_{i,j})$ corresponding to each of the terms of the sum in (2.3).

## The Heisenberg Algebra

To illustrate how the method from the previous section is applied, we use the Heisenberg Lie algebra to obtain duality functions for two stochastic processes. Let us first describe the processes.

The independent random walker process IRW is a Markov jump process where particles move independently between *N* sites, and each site can contain an arbitrary number of particles. Particles jump from site *i* to site *j* with rate proportional to the number of particles $n_i$ at site *i*. Let $p_{i,j}\ge 0$. The generator of this process is the difference operator acting on appropriate function in $F({\mathbb {N}}^N)$ given by

3.1 $$\begin{aligned} L^{\mathrm {IRW}}f(n)&= \sum _{1 \le i < j \le N} p_{i,j}\left[ n_i \left( f(n^{i,j}) - f(n) \right) +\, n_j \left( f(n^{j,i} - f(n) \right) \right] , \qquad n \in {\mathbb {N}}^N.\nonumber \\ \end{aligned}$$

Here $n^{i,j} = n+e_i-e_j$, where $e_i$ the standard basis vector with 1 as *i*th component and all other component are 0.

The second process is a Feller diffusion process on ${\mathbb {R}}^N$ with a constant diffusion matrix, and a drift vector which is a function of the difference of pairs of coordinates. It can be considered as *N* Brownian motions which are attracted to each other with a rate proportional to their distances. The generator is a differential operator on appropriate functions in $F({\mathbb {R}}^N)$ given by

3.2 $$\begin{aligned} L^{\mathrm {DIF}}f(x)&= c\sum _{1 \le i < j \le N} p_{i,j}\left[ \left( \frac{\partial }{\partial x_i}- \frac{ \partial }{\partial x_j} \right) ^2 f(x)\right. \nonumber \\&\quad \left. -\, (x_i-x_j)\left( \frac{\partial }{\partial x_i}- \frac{ \partial }{\partial x_j} \right) f(x) \right] , \end{aligned}$$

where $$x \in {\mathbb {R}}^N$$ and $$c>0$$.

Note that both generators have the form (2.1).

The Heisenberg algebra ${\mathfrak {h}}$ is the Lie algebra with generators $a,a^\dagger ,Z$ satisfying

3.3 $$\begin{aligned} {[a,Z]}=[a^\dagger ,Z]=0, \qquad [a^\dagger ,a]=Z. \end{aligned}$$

The $*$-structure is given by $a^*=a^\dagger$, $(a^\dagger )^*=a$ and $Z^*=Z$. ${\mathfrak {h}}$ has a representation $\rho _{c}$ with parameter $c>0$ on $F({\mathbb {N}})$ given by

3.4 $$\begin{aligned} \begin{aligned} {[\rho _{c}(a)f]}(n)&= n f(n-1), \\ {[\rho _{c}(a^\dagger )f]}(n)&= cf(n+1),\\ {[\rho _{c}(Z)f)]}(n)&= cf(n), \end{aligned} \end{aligned}$$

where $f(-1)=0$ by convention. Then $\rho _{c}$ is a $*$-representation on the weighted $L^2$-space ${\mathcal {H}}_c = \ell ^2({\mathbb {N}},w_c)$ consisting of functions in $F({\mathbb {N}})$ that have finite norm with respect to the inner product

$$\begin{aligned} \langle f,g\rangle = \sum _{n \in {\mathbb {N}}} w_c(n)\, f(n) \overline{g(n)}, \qquad w_c(n) = \frac{c^n}{n!}e^{-c}. \end{aligned}$$

$\rho _c$ is an unbounded representation, with dense domain the set $F_0({\mathbb {N}})$ consisting of finitely supported functions.

Define $$Y \in U({\mathfrak {h}})^{\otimes 2}$$ by

3.5 $$\begin{aligned} Y= (1 \otimes a - a \otimes 1 )(a^\dagger \otimes 1 - 1 \otimes a^\dagger ). \end{aligned}$$

This element gives us the relation with the system of independent random walkers.

### Lemma 3.1

For $c>0$, let $\rho$ be the tensor product representation $\rho = \rho _{c} \otimes \cdots \otimes \rho _{c}$ of ${\mathfrak {h}}$ on ${\mathcal {H}}_c^{\otimes N}$, then

$$\begin{aligned} L^{\mathrm {IRW}} = c^{-1}\sum _{1 \le i < j \le N} p_{i,j} \, \rho (Y_{i,j}). \end{aligned}$$

### Proof

It suffices to consider $(\rho _{c} \otimes \rho _{c}) (Y)$ acting on functions in two variables $n_1$ and $n_2$. From (3.4) and (3.5) we find

$$\begin{aligned} \begin{aligned}&{[\rho _{c}\otimes \rho _{c}(Y)f]}(n_1,n_2) \\&\quad = c n_1 \Big ( f(n_1-1,n_2+1) - f(n_1,n_2) \Big ) + c n_2 \Big ( f(n_1+1,n_2-1) - f(n_1,n_2) \Big ). \end{aligned} \end{aligned}$$

This corresponds to the term $(i,j)=(1,2)$ in (3.1). $\square$

### Remark 3.2

We can also consider the tensor product representation $\rho _{c_1} \otimes \cdots \otimes \rho _{c_N}$ with (possibly) $c_i \ne c_j$. This leads to a generator of a Markov process depending on *N* different parameters. However, to prove self-duality it seems crucial to assume $c_i=c$ for all *i*, see Lemma 3.5 later on.

### Charlier Polynomials and Self-Duality of IRW

The Charlier polynomials [11, Section 9.14] are defined by

$$\begin{aligned} C_n(x;c) = \,_{2}F_{0}\!\left( \genfrac{}{}{0.0pt}{}{-n,-x}{\text {--}} \,;-\frac{1}{c} \right) . \end{aligned}$$

They form an orthogonal basis for $\ell ^2({\mathbb {N}}, w_c)$, with orthogonality relations

$$\begin{aligned} \sum _{x\in {\mathbb {N}}}w_c(x)\, C_m(x;c) C_n(x;c) = \delta _{mn} c^{-n}n!, \qquad a>0, \end{aligned}$$

and they have the following raising and lowering property,

3.6 $$\begin{aligned} \begin{aligned} nC_{n-1}(x;c)&= cC_n(x;c) - c C_{n}(x+1;c), \\ cC_{n+1}(x;c)&=c C_n(x;c)- x C_{n}(x-1;c). \end{aligned} \end{aligned}$$

They are self-dual, i.e. $C_n(x;c)= C_x(n;c)$.

Let us consider the actions of *a* and $a^\dagger$ on the Charlier polynomials

$$\begin{aligned} C(n,x;c)= e^{c}C_n(x;c). \end{aligned}$$

The reason for this normalization is to obtain a unitary intertwiner with *C*(*n*, *x*; *c*) as a kernel later on. For notational convenience we will often omit the dependence on *c* in the notation; $C(n,x)=C(n,x;c)$. Using the raising and lowering properties (3.6) we obtain

3.7 $$\begin{aligned} \begin{aligned} {[\rho _c(a) C(\cdot ,x)](n)}&= n C(n-1,x) = c C(n,x) - c C(n,x+1),\\ {[\rho _c(a^\dagger ) C(\cdot ,x)](n)}&= c C(n+1,x) = c C(n,x) - x C(n,x-1). \end{aligned} \end{aligned}$$

Note that the actions on the *x*-variable are similar to the actions of $Z-a$ and $Z-a^\dagger$ in the *n*-variable. This motivates the definition of the following isomorphism.

#### Lemma 3.3

The assignments

$$\begin{aligned} \theta (a)=Z-a,\quad \theta (a^\dagger ) = Z-a^\dagger , \quad \theta (Z) = Z, \end{aligned}$$

extend uniquely to a Lie algebra isomorphism $\theta :{\mathfrak {h}} \rightarrow {\mathfrak {h}}$.

#### Proof

The proof consists of checking the commutation relations (3.3), which is a straightforward computation. $\square$

Note that by Lemma 3.3 and (3.7)

$$\begin{aligned} {[\rho _c(\theta (a)) C(\cdot ;x)]}(n) = c\,C(n,x+1), \qquad {[\rho _c(\theta (a^\dagger )) C(\cdot ;x)]}(n) = x\, C(n,x-1). \end{aligned}$$

Furthermore, $\theta$ preserves the $*$-structure, i.e. $\theta (X^*) = \theta (X)^*$. Clearly, $\rho _c\circ \theta$ is again a $*$-representation of ${\mathfrak {h}}$ on ${\mathcal {H}}_c$. We will use the Charlier polynomials to construct a unitary intertwiner between $\rho _c$ and $\rho _c \circ \theta$.

#### Proposition 3.4

Define the operator $\Lambda :F_0({\mathbb {N}})\rightarrow F({\mathbb {N}})$ by

$$\begin{aligned} (\Lambda f)(x) = \sum _{n \in {\mathbb {N}}} w_c(n) f(n) C(n,x;c), \end{aligned}$$

then $\Lambda$ extends to a unitary operator $\Lambda :{\mathcal {H}}_c \rightarrow {\mathcal {H}}_c$, and intertwines $\rho _c$ with $\rho _c\circ \theta$. Furthermore, the kernel *C*(*n*, *x*) satisfies

3.8 $$\begin{aligned} {[\rho _c(X^*) C(\cdot ,x)]}(n) = [\rho _c(\theta (X)) C(n,\cdot )](x), \qquad X \in {\mathfrak {h}}. \end{aligned}$$

#### Proof

The cheap duality functions $\delta _m(n) = \frac{ \delta _{m,n} }{w_c(n)}$ form an orthogonal basis for ${\mathcal {H}}_c$ with squared norm $\Vert \delta _m\Vert ^2 = \frac{1}{w_c(m)}$. Applying $\Lambda$ to $\delta _m$ gives

$$\begin{aligned} \Lambda (\delta _m)(x) = C(m,x). \end{aligned}$$

From the orthogonality relations for the Charlier polynomials we find that the squared norm of *C*(*m*, *x*) is $\Vert C(m,\cdot )\Vert ^2 = \frac{1}{w_c(m)}$. So $\Lambda$ maps an orthogonal basis to another orthogonal basis with the same norm, hence $\Lambda$ is unitary.

To apply Lemma 2.1 we need to verify that (3.8) is satisfied. It is enough to do this for $X = a, a^\dagger , Z$. Using (3.7) and $a^*=a^\dagger$ we see that

$$\begin{aligned} {[\rho _c(a^*) C(\cdot ,x)]}(n)= & {} c C(n,x) - x C(n,x-1) = [\rho _c(Z-a)C(n,\cdot )](x), \\ {[\rho _c((a^\dagger )^*) C(\cdot ,x)]}(n)= & {} c C(n,x) - c C(n,x+1) = {[\rho _c(Z-a^\dagger )C(n,\cdot )](x).} \end{aligned}$$

The action of *Z* is clear. Now the result follows from the definition of $\theta$, see Lemma 3.3.

$$\square $$

We are almost ready to prove self-duality for IRW, but first we need to know the image of *Y*, see (3.5), under the isomorphism $\theta \otimes \theta$.

#### Lemma 3.5

The following identity in $U({\mathfrak {h}})^{\otimes 2}$ holds:

$$\begin{aligned} \theta \otimes \theta (Y) = Y + R \end{aligned}$$

with

$$\begin{aligned} \begin{aligned} R =\,&1 \otimes Za^\dagger - Z \otimes a^\dagger + Za^\dagger \otimes 1 - a^\dagger \otimes Z + 1 \otimes aZ - Z \otimes a + aZ \otimes 1 - a \otimes Z\\&+ 2 \, Z \otimes Z - Z^2 \otimes 1 - 1 \otimes Z^2. \end{aligned} \end{aligned}$$

In the representation $\rho _c \otimes \rho _c$, the element *R* is the zero operator on ${\mathcal {H}}_c \otimes {\mathcal {H}}_c$.

#### Proof

After a somewhat tedious computation using the definition of $\theta$ in Lemma 3.3, we find the explicit expression for $\theta \otimes \theta (Y)$. Using $\rho _c(Z) = c\, \mathrm {Id}$ it follows that $\rho _c \otimes \rho _c (R) = 0$. $\square$

We can now apply Theorem 2.2 with $\rho =\rho _{c} \otimes \cdots \otimes \rho _{c}$ and $\sigma =\rho \circ (\theta \otimes \cdots \otimes \theta )$. Using Lemma 3.5 we find $\sigma (Y_{i,j})=\rho (Y_{i,j})$, and then it follows that $\sum p_{i,j} \sigma (Y_{i,j}) = L^{\mathrm {IRW}}$, see Lemma 3.1. So we obtain the well-known self-duality of the independent random walker process. Here the duality function is a product of Charlier polynomials.

#### Theorem 3.6

The operator $L^{\mathrm {IRW}}$ given by (3.1) is self-dual, with duality function

$$\begin{aligned} \prod _{j=1}^N C(n_j,x_j;c), \qquad c>0. \end{aligned}$$

#### Remark 3.7

The intertwining operator $\Lambda : {\mathcal {H}}_c \rightarrow {\mathcal {H}}_c$ maps the orthogonal basis of cheap duality functions to an orthogonal basis of Charlier polynomials (see the proof of Proposition 3.4), so $\Lambda$ can be considered as a change of basis for ${\mathcal {H}}_c$. In this sense the self-duality functions in Theorem 3.6 are the matrix elements of a change of base in ${\mathcal {H}}_c^{\otimes N}$.

### Hermite Polynomials and Duality Between IRW and the Diffusion Process

The Hermite polynomials [11, Section 9.15] are defined by

$$\begin{aligned} H_n(x) = (2x)^n \,_{2}F_{0}\!\left( \genfrac{}{}{0.0pt}{}{-\frac{n}{2}, -\frac{n-1}{2}}{\text {--}} \,;-\frac{1}{x^2} \right) . \end{aligned}$$

They form an orthogonal basis for $L^2({\mathbb {R}}, e^{-x^2} dx)$, with orthogonality relations

$$\begin{aligned} \frac{1}{\sqrt{\pi }}\int _{\mathbb {R}}H_m(x) H_n(x) e^{-x^2}\, dx = \delta _{mn} 2^n n!, \end{aligned}$$

and they have the following lowering and raising properties

3.9 $$\begin{aligned} \frac{d}{dx}H_n(x)= & {} 2n H_{n-1}(x), \nonumber \\ \left( -\frac{d}{dx}+2x\right) H_n(x)= & {} H_{n+1}(x). \end{aligned}$$

With the lowering and raising operators for the Hermite polynomials we can realize *a* and $a^\dagger$ as differential operators. We define

$$\begin{aligned} H(n,x;c) = e^{\frac{c}{2}} (2c)^{-\frac{n}{2}} H_n\left( \tfrac{x}{\sqrt{2c}} \right) . \end{aligned}$$

Using the representation $\rho$ (3.4) and the differential operators (3.9) we find the following result.

#### Lemma 3.8

The Hermite polynomials $H(n,x)=H(n,x;c)$ satisfy

$$\begin{aligned} {[\rho _c(a)H(\cdot ,x)]}(n)= & {} c\frac{\partial }{\partial x} H(n,x),\\ {[\rho _c(a^\dagger ) H(\cdot ,x)]}(n)= & {} \left( x-c \frac{\partial }{\partial x} \right) H(n,x). \end{aligned}$$

Next we define an unbounded $*$-representation $\sigma _c$ of ${\mathfrak {h}}$ on the Hilbert space $\mathrm H_c= L^2({\mathbb {R}}, w(x;c)dx)$, where

$$\begin{aligned} w(x;c) = \frac{e^{-\frac{x^2}{2c}}}{\sqrt{2c\pi }}. \end{aligned}$$

The Hermite polynomials *H*(*n*, *x*) form an orthogonal basis for $\mathrm H_c$, with squared norm $\Vert H(n,\cdot )\Vert ^2 = \frac{1}{w_c(n)}$. We define the representation $\sigma _c$ by

$$\begin{aligned} \begin{aligned} {[\sigma _c(a) f](x)}&= xf(x) - c \frac{\partial }{\partial x}f(x),\\ {[\sigma _c(a^\dagger ) f]}(x)&= c \frac{ \partial }{\partial x}f(x),\\ {[\sigma _c(Z)f](x)}&= cf(x). \end{aligned} \end{aligned}$$

As a dense domain we take the set of polynomials ${\mathcal {P}}$.

#### Proposition 3.9

Define $$\Lambda :F_0({\mathbb {N}})\rightarrow F({\mathbb {R}})$$ by

$$\begin{aligned} (\Lambda f)(x) = \sum _{n \in {\mathbb {N}}} w_c(n) f(n) H(n,x;c), \end{aligned}$$

then $\Lambda$ extends to a unitary operator $\Lambda :{\mathcal {H}}_c \rightarrow \mathrm H_c$ intertwining $\rho _c$ with $\sigma _c$. Furthermore, the kernel *H*(*n*, *x*) satisfies

$$\begin{aligned} {[\rho _c(X^*)H(\cdot ,x)]}(n) = [\sigma _c(X) H(n,\cdot )](x), \qquad X \in {\mathfrak {h}}. \end{aligned}$$

#### Proof

Unitarity of $\Lambda$ is proved in the same way as in Proposition 3.4. The intertwining property for the kernel follows from Lemma 3.8. Lemma 2.1 then shows that $\Lambda$ intertwines $\rho _c$ and $\sigma _c$. $\square$

Similar as in Lemma 3.1 we find that the generator $L^{\mathrm {DIF}}$ defined by (3.2) is the realization of *Y* defined by (3.5) on the Hilbert space $\mathrm H_{c}^{\otimes N}$.

#### Lemma 3.10

For $c >0$ define $\sigma = \sigma _{c}\otimes \cdots \otimes \sigma _{c}$, then

$$\begin{aligned} L^{\mathrm {DIF}} = c^{-1}\sum _{1 \le i<j\le N} p_{i,j}\, \sigma _c(Y_{i,j}). \end{aligned}$$

Finally, applying Theorem 2.2 we obtain duality between $L^{\mathrm {IRW}}$ and $L^{\mathrm {DIF}}$, with duality function given by Hermite polynomials.

#### Theorem 3.11

$L^{\mathrm {IRW}}$ and $L^{\mathrm {DIF}}$ are in duality, with duality function given by

$$\begin{aligned} \prod _{j=1}^N H(n_j,x_j;c). \end{aligned}$$

#### Remark 3.12

This duality between $L^{\mathrm {IRW}}$ and $L^{\mathrm {DIF}}$ was also obtained in [7, Remark 3.1], but Hermite polynomials are not mentioned there. Hermite polynomials of even degree have appeared as duality functions in [5, §4.1.1]; this can be considered as a special case of duality involving Laguerre polynomials, see [5, §4.2.1] or Theorem 4.15.

### The Exponential Function and Self-Duality of the Diffusion Process

To show self-duality of the differential operator $L^{\mathrm {DIF}}$, the following isomorphism is useful.

#### Lemma 3.13

The assignments

$$\begin{aligned} \theta (a) = \frac{1}{2}(a-a^\dagger ), \qquad \theta (a^\dagger ) = i(a+a^\dagger ), \qquad \theta (Z) = iZ, \end{aligned}$$

extend uniquely to a Lie algebra isomorphism $\theta :{\mathfrak {h}} \rightarrow {\mathfrak {h}}$.

#### Proof

We just need to check commutation relations, which is a direct calculation. $\square$

Observe that in the representation $\sigma _c$, $\theta (a)$ and $\theta (a^\dagger )$ are the operators

$$\begin{aligned} \sigma _c(\theta (a)) = \frac{x}{2}-c\frac{\partial }{\partial x}, \qquad \sigma _c(\theta (a^\dagger )) = ix. \end{aligned}$$

The kernel of the (yet to be defined) intertwining operator is the exponential function

$$\begin{aligned} \phi (x,y;c) = \exp \left( \frac{x^2+y^2}{4c}-\frac{ixy}{c}\right) , \qquad x,y \in {\mathbb {R}}. \end{aligned}$$

#### Lemma 3.14

The function $\phi (x,y)=\phi (x,y;c)$ satisfies

$$\begin{aligned} \begin{aligned} {[\sigma _c(\theta (a)) \phi (\cdot ,y)](x)}&= iy\, \phi (x,y),\\ {[\sigma _c(\theta (a^\dagger )) \phi (\cdot ,y)](x)}&= \left( \frac{y}{2}-c\frac{\partial }{\partial y}\right) \phi (x,y), \end{aligned} \end{aligned}$$

#### Proof

From

$$\begin{aligned} c \frac{\partial }{\partial x}\phi (x,y) = \left( \frac{x}{2}-iy\right) \phi (x,y) \end{aligned}$$

we obtain

$$\begin{aligned}{}[\sigma _c(\theta (a)) \phi (\cdot ,y)](x) = \left( \frac{x}{2}-c\frac{\partial }{\partial x}\right) \phi (x,y) = iy\, \phi (x,y). \end{aligned}$$

Using symmetry in *x* and *y*, we find

$$\begin{aligned} {[\sigma _c(\theta (a^\dagger )) \phi (\cdot ,y)]}(x) = ix\,\phi (x,y) =\left( \frac{y}{2}-c\frac{\partial }{\partial y}\right) \phi (x,y). \end{aligned}$$

$$\square $$

Now we can show that the integral operator with $\phi$ as a kernel is the desired intertwining operator.

#### Proposition 3.15

Define $$\Lambda :{\mathcal {P}}\rightarrow F({\mathbb {R}})$$ by

$$\begin{aligned} (\Lambda f)(y) = \int _{\mathbb {R}}f(x) \phi (x,y;c) w(x;c)\, dx, \end{aligned}$$

then $\Lambda$ extends to a unitary operator $\Lambda : \mathrm H_c \rightarrow \mathrm H_c$ intertwining $\sigma _c$ with $\sigma _c$. Furthermore, the kernel $\phi (x,y)$ satisfies

$$\begin{aligned} {[\sigma _c(X^*)\phi (\cdot ,y)]}(x) = [\sigma _c(X) \phi (x,\cdot )](y), \qquad X \in {\mathfrak {h}}. \end{aligned}$$

#### Proof

Unitarity of $\Lambda$ follows from unitarity of the Fourier transform. From Lemma 3.14 we find

$$\begin{aligned} {[\sigma _c(\theta (X^*))\phi (\cdot ,y)]}(x) = [\sigma _c(\theta (X)) \phi (x,\cdot )](y), \qquad X \in {\mathfrak {h}}, \end{aligned}$$

which proves the result, since $\theta$ is an isomorphism. $\square$

By applying Theorem 2.2 and using Lemma 3.10 we obtain self-duality of $L^{\mathrm {DIF}}$ (3.2).

#### Theorem 3.16

$L^{\mathrm {DIF}}$ is self-dual, with duality function given by

$$\begin{aligned} \prod _{j=1}^N \phi (x_j,y_j;c). \end{aligned}$$

## The Lie Algebra $\varvec{\mathfrak {su}}(1,1)$

In this section we use the Lie algebra $\mathfrak {su}(1,1)$ to find duality functions for two stochastic processes. The first one is the symmetric inclusion process SIP(*k*), $k \in {\mathbb {R}}_{>0}^N$, which is a Markov jump process on *N* sites, where each site can contain an arbitrary number of particles. Jumps between two sites, say *i* and *j*, occur at a rate proportional to the number of particles $n_i$ and $n_j$. Let $p_{i,j}\ge 0$. The generator of this process is given by

4.1 $$\begin{aligned} L^{\mathrm {SIP}} f(n)&= \sum _{1 \le i < j \le N} p_{i,j} \left[ n_i(2k_j+n_j) \left( f(n^{i,j}) - f(n) \right) \right. \nonumber \\&\quad \left. +\, n_j(2k_i+n_i) \left( f(n^{j,i}) - f(n) \right) \right] , \end{aligned}$$

with $$n=(n_1,\ldots ,n_N) \in {\mathbb {N}}^N$$.

The second process is the Brownian energy process BEP(*k*), $k \in {\mathbb {R}}_{>0}^N$, which is a Markov diffusion process that describes the evolution of a system of *N* particles that exchange energies. The energy of particle *i* is $x_i>0$. The generator is given by

4.2 $$\begin{aligned} L^{\mathrm {BEP}} f(x)&= \sum _{1 \le i<j \le N} p_{i,j} \left[ x_i x_j \left( \frac{\partial }{\partial x_i} - \frac{\partial }{\partial x_j} \right) ^2f(x) \right. \nonumber \\&\quad \left. -\, 2 (k_i x_i - k_j x_j) \left( \frac{\partial }{\partial x_i} - \frac{\partial }{\partial x_j} \right) f(x) \right] , \end{aligned}$$

with $$x=(x_1,\ldots ,x_N) \in {\mathbb {R}}_{>0}^N$$.

The Lie algebra $\mathfrak {sl}(2,{\mathbb {C}})$ is generated by *H*, *E*, *F* with commutation relations

$$\begin{aligned}{}[H,E]=2E, \quad [H,F]=-2F, \quad [E,F]=H. \end{aligned}$$

The Lie algebra $\mathfrak {su}(1,1)$ is $\mathfrak {sl}(2,{\mathbb {C}})$ equipped with the $*$-structure

$$\begin{aligned} H^*=H, \quad E^*=-F, \quad F^*=-E. \end{aligned}$$

The Casimir element $\Omega$ is a central self-adjoint element of the universal enveloping algebra $U\big (\mathfrak {su}(1,1)\big )$ given by

4.3 $$\begin{aligned} \Omega = \frac{1}{2}H^2 + EF + FE. \end{aligned}$$

Note that $$\Omega ^*=\Omega $$.

We consider the following representation of $\mathfrak {su}(1,1)$. Let $k>0$ and $0<c<1$. The representation space is the weighted $L^2$-space ${\mathcal {H}}_{k,c}=\ell ^2({\mathbb {N}},w_{k,c})$ consisting of functions on ${\mathbb {N}}$ which have finite norm with respect to the inner product

$$\begin{aligned} \langle f,g\rangle = \sum _{n \in {\mathbb {N}}} w_{k,c}(n)\, f(n) \overline{g(n)}, \qquad w_{k,c}(n) = \frac{ (2k)_n }{n!}c^n (1-c)^{2k}. \end{aligned}$$

The actions of the generators are given by

4.4 $$\begin{aligned} \begin{aligned} {[\pi _{k,c}(H) f]}(n) =&\ 2(k+n) f(n), \\ {[\pi _{k,c}(E)f]} (n) =&\ \frac{n}{\sqrt{c}} f(n-1), \\ {[\pi _{k,c}(F)f]} (n) =&\ -\sqrt{c}\,(2k+n) f(n+1), \end{aligned} \end{aligned}$$

where $f(-1)=0$ by convention. This defines an unbounded $*$-representation on ${\mathcal {H}}_{k,c}$, with dense domain $F_0({\mathbb {N}})$. Note that $[\pi _{k,c}(\Omega ) f](n) = 2k(k-1) f(n)$.

### Remark 4.1

For $0<c_1,c_2<1$ define a unitary operator $I:{\mathcal {H}}_{k,c_1}\rightarrow {\mathcal {H}}_{k,c_2}$ by

$$\begin{aligned} (If)(n) = \left( \frac{c_1}{c_2}\right) ^{n/2} f(n). \end{aligned}$$

Then $I\circ \pi _{k,c_1} = \pi _{k,c_2} \circ I$, so for fixed $k>0$ all representations $\pi _{k,c}$, $0<c<1$, are unitarily equivalent (we can even take $c \ge 1$ if we omit the factor $(1-c)^{2k}$ from the weight function $w_{k,c}$). From here on we assume that *c* is a fixed parameter, and just write $\pi _k$ and ${\mathcal {H}}_k$ instead of $\pi _{k,c}$ and ${\mathcal {H}}_{k,c}$.

The generator $L^{\mathrm {SIP}}$ is related to the Casimir $\Omega$. Recall that the coproduct $\Delta$ is given by $\Delta (X) = 1\otimes X + X \otimes 1$, and $\Delta$ extends as an algebra morphism to $U(\mathfrak {su}(1,1))$. This gives

$$\begin{aligned} \Delta (\Omega ) = 1\otimes \Omega + \Omega \otimes 1 + H \otimes H + 2F \otimes E + 2E \otimes F. \end{aligned}$$

We set

4.5 $$\begin{aligned} Y = \frac{1}{2}\Big (1\otimes \Omega + \Omega \otimes 1 - \Delta (\Omega )\Big ). \end{aligned}$$

The relation to the symmetric inclusion process is as follows.

### Lemma 4.2

For $k=(k_1,\ldots ,k_N) \in {\mathbb {R}}_{>0}^N$ define $\pi _k = \pi _{k_1}\otimes \cdots \otimes \pi _{k_N}$, then

$$\begin{aligned} L^{\mathrm {SIP}} =\sum _{1 \le i<j \le N} p_{i,j} \,\big [\pi _k (Y_{i,j}) + 2k_ik_j \big ] \end{aligned}$$

### Proof

Consider $L_{1,2}= \pi _{k_1}\otimes \pi _{k_2}(Y)+2k_1k_2$. It suffices to show that $L_{1,2}$ gives the term $(i,j) = (1,2)$ in (4.1). From (4.5) and (4.4) we find that $L_{1,2}$ acts on $f \in {\mathcal {H}}_{k_1} \otimes {\mathcal {H}}_{k_2}$ by

$$\begin{aligned} {[L_{1,2} f]}(n_1,n_2)&= n_1(2k_2+n_2) [f(n_1-1,n_2+1) - f(n_1,n_2)] \\&\quad +\, n_2(2k_1+n_1) [f(n_1+1,n_2-1) - f(n_1,n_2)], \end{aligned}$$

which is the required expression. $\square$

In order to obtain duality functions we consider eigenfunctions of the following self-adjoint element as in [12]:

4.6 $$\begin{aligned} X_a = -a H + E - F \in \mathfrak {su}(1,1), \qquad a \in {\mathbb {R}}. \end{aligned}$$

Depending on the value of *a* this is either an elliptic element ($|a|>1$), parabolic element ($|a|=1$), or hyperbolic element ($|a|<1$), corresponding to the associated one-parameter subgroups in $\mathrm {SU}(1,1)$.

### Meixner Polynomials and Self-Duality for SIP

The Meixner polynomials [11, Section 9.10] are defined by

4.7 $$\begin{aligned} M_n(x;\beta ,c) = \,_{2}F_{1}\!\left( \genfrac{}{}{0.0pt}{}{-n,-x}{\beta } \,;1-\frac{1}{c} \right) . \end{aligned}$$

These are self-dual: $M_n(x;\beta ,c) = M_x(n;\beta ,c)$ for $x \in {\mathbb {N}}$. For $\beta >0$ and $0<c<1$, the Meixner polynomials are orthogonal with respect to a positive measure on ${\mathbb {N}}$,

$$\begin{aligned} \sum _{x=0}^\infty \frac{(\beta )_x c^x}{ x! } M_m(x) M_n(x) = \delta _{mn} \frac{ c^{-n} n!}{(\beta )_n (1-c)^\beta }, \end{aligned}$$

and the polynomials form a basis for the corresponding Hilbert space. The three-term recurrence relation for the Meixner polynomials is

$$\begin{aligned} (c-1)(x+\tfrac{1}{2}\beta ) M_n(x) = c(n+\beta ) M_{n+1}(x) - (c+1)(n+\tfrac{1}{2}\beta ) M_n(x) + n M_{n-1}(x). \end{aligned}$$

Using the self-duality this also gives a difference equation in the *x*-variable for the Meixner polynomials.

We set $a(c) = \frac{1+c}{2\sqrt{c}}$, so that $a(c)>1$. The action of

$$\begin{aligned} X_{a(c)}= - \frac{1+c}{2\sqrt{c}}H+E-F \end{aligned}$$

on $f \in {\mathcal {H}}_{k}$ is given by

$$\begin{aligned} {[\pi _k(X_{a(c)}) f]}(n) = \sqrt{c}(2k+n) f(n+1) - \frac{1+c}{\sqrt{c}} (k+n) f(n) + \frac{n}{\sqrt{c}} f(n-1). \end{aligned}$$

Eigenfunctions can be given in terms of the Meixner polynomials

$$\begin{aligned} M(n,x;k,c) = M_n(x;2k,c). \end{aligned}$$

#### Lemma 4.3

The Meixner polynomials $M(n,x)=M(n,x;k,c)$ are eigenfunctions of $\pi _k(X_{a(c)})$, i.e.

$$\begin{aligned} {[\pi _k(X_{a(c)}) M(\cdot ,x)]}(n) = \frac{c-1}{\sqrt{c}}(x+k)\, M(n,x), \qquad x \in {\mathbb {N}}. \end{aligned}$$

#### Proof

This follows from the three-term recurrence relation for the Meixner polynomials.

$$\square $$

Using the difference equation for the Meixner polynomials, we can realize *H* as a difference operator acting on *M*(*n*, *x*) in the *x*-variable.

#### Lemma 4.4

The following identity holds:

$$\begin{aligned} \left[ \pi _k(H) M(\cdot ,x)\right] (n)&= -\frac{2c}{1-c} (2k+x) M(n,x+1) + \frac{1+c}{1-c} 2(k+x) M(n,x) \\&\quad -\,\frac{2x}{1-c} M(n,x-1). \end{aligned}$$

#### Proof

We use the difference equation for *M*(*n*, *x*), which is, by self-duality, equivalent to the three-term recurrence relation:

$$\begin{aligned} (c-1) (n+k) \, M(n,x) = c (2k+x) M(n,x+1) - (1+c)(k+x) M(n,x) + x M(n,x-1). \end{aligned}$$

Since $[\pi _k(H)M(\cdot ,x)](n) = 2(k+n) M(n,x)$ the result follows. $\square$

With the actions of $X_{a(c)}$ and *H* on Meixner polynomials, it is possible to express *E* and *F* acting on *M*(*n*, *x*) as three-term difference operators in the variable *x*. This leads to a representation by difference operators in *x*, in which the basis elements *H*, *E* and *F* all act by three-term difference operators. Having actions of *H*, *E* and *F*, we can express, after a large computation, $\Delta (\Omega )$ in terms of difference operators in two variables $x_1$ and $x_2$. We prefer, however, to work with a simpler representation in which *H* acts as a multiplication operator, and *E* and *F* as one-term difference operators. Note that the action of $X_{a(c)}$ in the *x*-variable corresponds up to a constant to the action of *H* in the *n*-variable, i.e. it is a multiplication operator. We can make a new $\mathfrak {sl}(2,{\mathbb {C}})$-triple with $X_{a(c)}$ playing the role of *H*. The following result from [8, §3.2], where it is proved using conjugation with a group element, gives the corresponding isomorphism.

#### Lemma 4.5

Define elements $H_c, E_c, F_c \in \mathfrak {su}(1,1)$ by

$$\begin{aligned} \begin{aligned} H_c&= \frac{1+c}{1-c}H - \frac{2\sqrt{c}}{1-c}E + \frac{2\sqrt{c}}{1-c} F, \\ E_c&= \frac{\sqrt{c}}{1-c} H - \frac{1}{1-c} E + \frac{c}{1-c}F,\\ F_c&= -\frac{\sqrt{c}}{1-c} H + \frac{c}{1-c} E - \frac{1}{1-c} F, \end{aligned} \end{aligned}$$

then the assignments

$$\begin{aligned} \theta _c(H) = H_c, \quad \theta _c(E)=E_c, \quad \theta _c(F) = F_c, \end{aligned}$$

extend to a Lie algebra isomorphism $\theta _c: \mathfrak {su}(1,1) \rightarrow \mathfrak {su}(1,1)$ with inverse $(\theta _c)^{-1} = \theta _{c }$. Furthermore, $\theta _c(\Omega ) = \Omega$.

#### Proof

To show that $\theta _c$ defines a Lie algebra homomorphism we need to check the commutation relations, which is a straightforward computation. The matrix of $\theta _c$ with respect to the basis $\{H,E,F\}$ is

$$\begin{aligned} \frac{1}{1-c} \begin{pmatrix} 1+c &{}-2\sqrt{c}&{}2\sqrt{c}\\ \sqrt{c} &{} - 1 &{} c \\ -\sqrt{c} &{} c &{} - 1 \end{pmatrix}. \end{aligned}$$

To show that $(\theta _c)^{-1} = \theta _c$, or equivalently $(\theta _c)^2 = \mathrm {Id}$, it suffices to check that the square of this matrix is the identity matrix. Again, this is a straightforward calculation. $\square$

Note that $\theta _c$ preserves the $*$-structure, i.e. $\theta _c(X^*) = \theta _c(X)^*$.

Observe that $H_c = \frac{2\sqrt{c}}{c-1}X_{a(c)}$, so we defined $H_c$ in such a way that

$$\begin{aligned} {[\pi _k(H_c) M(\cdot ,x)]}(n) = 2(k+x) M(n,x). \end{aligned}$$

By Lemma 4.5,

$$\begin{aligned} H = (\theta _c)^{-1}(H_c) = \theta _{c}(H_c) = \frac{1+c}{1-c}H_c - \frac{2\sqrt{c}}{1-c}E_c + \frac{2\sqrt{c}}{1-c} F_c. \end{aligned}$$

This gives

4.8 $$\begin{aligned} E_c-F_c= & {} \frac{ 1+c}{2\sqrt{c}}H_c-\frac{1-c}{2\sqrt{c}} H ,\nonumber \\ E_c+F_c= & {} \frac{1-c}{4\sqrt{c}} [H,H_c], \end{aligned}$$

which shows that we can express $E_c$ and $F_c$ completely in terms of $H_c$ and *H*. This allows us to write down explicit actions of $E_c$ and $F_c$ acting on *M*(*n*, *x*) in the *x*-variable. This then shows that *M*(*n*, *x*) has the desired intertwining properties.

#### Lemma 4.6

The functions *M*(*n*, *x*) satisfy

$$\begin{aligned} \begin{aligned} {[\pi _k(H_c)M(\cdot ,x)]}(n)&= 2(k+x) M(n,x),\\ {[\pi _k(E_c)M(\cdot ,x)]}(n)&=\sqrt{c}(2k+x) M(n,x-1),\\ {[\pi _k(F_c)M(\cdot ,x)]}(n)&= -\frac{x}{\sqrt{c}}M(n,x+1). \end{aligned} \end{aligned}$$

#### Proof

We already know the action of $H_c$. Using (4.8) and Lemma 4.4 for the action of *H* we find

$$\begin{aligned} \begin{aligned} \left[ \pi _k(E_c-F_c) M(\cdot ,x)\right] (n)&= \sqrt{c}(2k+x) M(n,x+1) + \frac{x}{\sqrt{c}} M(n,x-1),\\ \left[ \pi _k(E_c+F_c) M(\cdot ,x)\right] (n)&= \sqrt{c}(2k+x) M(n,x+1) - \frac{x}{\sqrt{c}} M(n,x-1), \end{aligned} \end{aligned}$$

which gives the actions of $E_c$ and $F_c$. $\square$

Now we are ready to define the intertwiner.

#### Proposition 4.7

The operator $\Lambda :F_0({\mathbb {N}}) \rightarrow F({\mathbb {N}})$ defined by

$$\begin{aligned} (\Lambda f)(x) = \sum _{n \in {\mathbb {N}}} w_k(n) f(n) M(n,x) \end{aligned}$$

extends to a unitary operator $\Lambda :{\mathcal {H}}_k \rightarrow {\mathcal {H}}_k$, and intertwines $\pi _k$ with $\pi _k\circ \theta _{c}$. Furthermore, the kernel *M*(*n*, *x*) satisfies

$$\begin{aligned} {[\pi _k(X^*) M(\cdot ,x)]}(n) = [\pi _k(\theta _{c}(X))M(n,\cdot )](x), \qquad X \in U(\mathfrak {su}(1,1)). \end{aligned}$$

#### Proof

Unitarity follows from the orthogonality relations and completeness of the Meixner polynomials. The properties of the kernel follow from Lemma 4.6. $\square$

For $j=1,\ldots ,N$ assume $k_j>0$ . From Proposition 4.7 we find unitary equivalence for tensor product representations,

$$\begin{aligned} \pi _{k_1} \otimes \cdots \otimes \pi _{k_N} \simeq (\pi _{k_1} \otimes \cdots \otimes \pi _{k_N}) \circ (\theta _{c} \otimes \cdots \otimes \theta _{c}). \end{aligned}$$

Using $\theta _{c}\otimes \theta _c \circ \Delta = \Delta \circ \theta _c$ and $\theta _c(\Omega ) = \Omega$, we see that, as in Lemma 4.2, the right-hand-side applied to $\sum p_{i,j}[Y_{i,j}-2k_ik_j]$ is the generator $L^{\mathrm {SIP}}$. Applying Theorem 2.2 then leads to self-duality of $L^{\mathrm {SIP}}$ (4.1), i.e. self-duality of the symmetric inclusion process SIP(*k*).

#### Theorem 4.8

The operator $L^{\mathrm {SIP}}$ defined by (4.1) is self-dual, with duality function

$$\begin{aligned} \prod _{j=1}^N M(n_j,x_j;k_j,c). \end{aligned}$$

#### Remark 4.9

The Lie algebra $\mathfrak {su}(2)$ is $\mathfrak {sl}(2,{\mathbb {C}})$ equipped with the $*$-structure defined by $H^*=H, E^*=F$. It is well known that $\mathfrak {su}(2)$ has only finite dimensional irreducible $*$-representations. These can formally be obtained from the $\mathfrak {su}(1,1)$ discrete series representation (4.4) by setting $k=-j/2$ for some $j \in {\mathbb {N}}$, where $j+1$ is the dimension of the corresponding representation space. If we make the corresponding substitution $k_i=-j_i/2$ in the generator (4.1) of the symmetric inclusion process, we obtain the generator of the symmetric exclusion process SEP on *N* sites where site *i* can have at most $j_i$ particles. Making a similar substitution in Theorem 4.8 we find self-duality of SEP, with duality function given by a product of Krawtchouk polynomials.

### Laguerre Polynomials and Duality Between SIP and BEP

The Laguerre polynomials [11, Section 9.12] are defined by

$$\begin{aligned} L_n^{(\alpha )}(x) = \frac{(\alpha +1)_n }{n!} \,_{1}F_{1}\!\left( \genfrac{}{}{0.0pt}{}{-n}{\alpha +1} \,;x \right) . \end{aligned}$$

They form an orthogonal basis for $L^2([0,\infty ), x^\alpha e^{-x}dx)$, with orthogonality relations given by

$$\begin{aligned} \int _0^\infty L_m^{(\alpha )}(x) L_n^{(\alpha )}(x) x^\alpha e^{-x} dx = \delta _{mn} \frac{ \Gamma (\alpha +n+1)}{n!}, \qquad \alpha >-1. \end{aligned}$$

The three-term recurrence relation is

$$\begin{aligned} -x L_n^{(\alpha )}(x) = (n+1) L_{n+1}^{(\alpha )}(x) - (2n+\alpha +1) L_n^{(\alpha )}(x) + (n+\alpha ) L_{n-1}^{(\alpha )}(x), \end{aligned}$$

and the differential equation is

$$\begin{aligned} x \frac{d^2}{dx^2} L_n^{(\alpha )}(x) + (\alpha +1-x) \frac{d}{dx} L_n^{(\alpha )}(x) = -n L_n^{(\alpha )}(x). \end{aligned}$$

We consider the action of the parabolic Lie algebra element $X_1 = -H+E-F$,

$$\begin{aligned} {[\pi _k(X_1) f]}(n) = -2(n+k) f(n) + \frac{n}{\sqrt{c}} f(n-1)+ \sqrt{c}(2k+n) f(n+1). \end{aligned}$$

Using the three-term recurrence relation for the Laguerre polynomials

$$\begin{aligned} L(n,x;k)=\frac{n! c^{-\frac{n}{2}}}{(2k)_n}L_n^{(2k-1)}(x), \end{aligned}$$

we find the following result.

#### Lemma 4.10

The Laguerre polynomials $L(n,x) = L(n,x;k)$ are eigenfunctions of $\pi _k(X_1)$,

$$\begin{aligned} {[\pi _k(X_1) L(\cdot ,x)]}(n) = -x L(n,x), \qquad x \in [0,\infty ). \end{aligned}$$

Just as we did in the elliptic case, we can define an algebra isomorphism that will be useful. In this case, the element $X_1$ corresponds to the generator *E*.

#### Lemma 4.11

The assignments

$$\begin{aligned} \begin{aligned} \theta (H)=E+F, \qquad \theta (E)=\frac{i}{2}(-H+E-F), \qquad \theta (F)=\frac{i}{2}(H+E-F), \end{aligned} \end{aligned}$$

extend to a Lie algebra isomorphism $\theta :\mathfrak {sl}(2,{\mathbb {C}}) \rightarrow \mathfrak {sl}(2,{\mathbb {C}})$. Furthermore, $\theta (\Omega )=\Omega$.

Note that $\theta (E)=\frac{i}{2}X_1$. Furthermore, $\theta$ does not preserve the $\mathfrak {su}(1,1)$-$*$-structure, i.e. $\theta (X^*) \ne \theta (X)^*$ in general. However, we can define another $*$-structure on $\mathfrak {sl}(2,{\mathbb {C}})$ by $\star = \theta ^{-1} \circ * \circ \theta$, then

4.9 $$\begin{aligned} H^\star = -H, \qquad E^\star = - E, \qquad F^\star = - F, \end{aligned}$$

which is the $*$-structure of $i\mathfrak {sl}(2,{\mathbb {R}})$. Next we determine the actions of the generators on the Laguerre polynomials.

#### Lemma 4.12

The Laguerre polynomials *L*(*n*, *x*) satisfy

$$\begin{aligned} \begin{aligned} {[\pi _k(\theta (H)) L(\cdot ,x)]}(n)&= 2x \frac{\partial }{\partial x} L(n,x) + (2k-x)\, L(n,x),\\ {[\pi _k(\theta (E)) L(\cdot ,x)]}(n)&= -\frac{1}{2} ix\, L(n,x),\\ {[\pi _k(\theta (F)) L(\cdot ,x)]}(n)&= -2ix \frac{\partial ^2}{\partial x^2} L(n,x) - 2i(2k-x) \frac{\partial }{\partial x} L(n,x) +\frac{i}{2}(4k-x) \,L(n,x). \end{aligned} \end{aligned}$$

#### Proof

The action of $\theta (E)$ is Lemma 4.10. From the differential equation for Laguerre polynomials we find

$$\begin{aligned} {[\pi _k(H)L(\cdot ,x)]}(n) = 2(k+n) L(n,x)= & {} -2x \frac{\partial ^2}{\partial x^2} L(n,x) \\&- 2(2k-x) \frac{\partial }{\partial x} L(n,x) +2k \,L(n,x). \end{aligned}$$

By linearity $\pi _k(H)$ extends to a differential operator acting on polynomials. Then the action of $\theta (H)$ is obtained from the identity $\theta (H)=E+F= -\frac{1}{2}[X_1,H]$. Finally, the action of $\theta (F)$ follows from $\theta (F) = \theta (E) + iH$. $\square$

Next we define an unbounded representation $\sigma _k$ of $\mathfrak {sl}(2,{\mathbb {C}})$ on $\mathrm H_k \!\!=\! L^2([0,\infty ), w(x;k)dx)$, where

$$\begin{aligned} w(x;k) = \frac{ x^{2k-1}e^{-x} }{\Gamma (2k)}. \end{aligned}$$

As a dense domain we take the set of polynomials ${\mathcal {P}}$. The representation $\sigma _k$ is defined on the generators *H*, *E*, *F* by

4.10 $$\begin{aligned} \begin{aligned} {[\sigma _k(H) f]}(x)&= 2x \frac{\partial }{\partial x}f(x) + (2k-x) f(x), \\ {[\sigma _k(E) f]}(x)&= -\frac{1}{2}ix f(x),\\ {[\sigma _k(F) f]}(x)&= -2ix \frac{\partial ^2}{\partial x^2} f(x) - 2i(2k-x) \frac{\partial }{\partial x} f(x) +\frac{i}{2}(4k-x) f(x). \end{aligned} \end{aligned}$$

Note that this is not a $*$-representation of $\mathfrak {su}(1,1)$, but $\sigma _k \circ \theta ^{-1}$ is. Equivalently, $\sigma _k$ is a $*$-representation on $\mathrm H_k$ with respect to the $*$-structure defined by (4.9). In the following lemma we give the intertwiner between $\pi _k$ and $\sigma _k\circ \theta ^{-1}$. The proof uses Lemma 4.12 and orthogonality and completeness of the Laguerre polynomials.

#### Proposition 4.13

The operator $\Lambda :F_0({\mathbb {N}}) \rightarrow F([0,\infty ))$ defined by

$$\begin{aligned} (\Lambda f)(x) = \sum _{n \in {\mathbb {N}}} w_k(n) f(n) L(n,x), \end{aligned}$$

extends to a unitary operator $\Lambda :{\mathcal {H}}_k \rightarrow \mathrm H_k$ intertwining $\pi _k$ with $\sigma _k\circ \theta ^{-1}$. Furthermore, the kernel *L*(*n*, *x*) satisfies

$$\begin{aligned} {[\pi _k(X^*) L(\cdot ,x)]}(n) = [ \sigma _k(\theta ^{-1}(X)) L(n,\cdot )](x), \qquad X \in U(\mathfrak {su}(1,1)). \end{aligned}$$

For $j=1,\ldots ,N$ let $k_j>0$, and define

4.11 $$\begin{aligned} \sigma _k = (\sigma _{k_1} \otimes \cdots \otimes \sigma _{k_N})\circ (\theta ^{-1} \otimes \cdots \otimes \theta ^{-1}), \end{aligned}$$

which is a $*$-representation of $\mathfrak {su}(1,1)$ on $\bigotimes _{j=1}^n H_{k_j}$. The counterpart of Lemma 4.2 for the representation $\sigma _k$ is as follows.

#### Lemma 4.14

The generator $L^{\mathrm {BEP}}$ given by (4.2) satisfies

$$\begin{aligned} L^{\mathrm {BEP}} = \sum _{1 \le i< j \le N} p_{i,j} \, \big [ \sigma _k(Y_{i,j}) + 2k_ik_j \big ], \end{aligned}$$

where *Y* is given by (4.5).

#### Proof

Using $\theta \otimes \theta \circ \Delta = \Delta \circ \theta$ and $\theta (\Omega )=\Omega$, we see that $(\theta \otimes \theta )(Y)=Y$. It is enough to calculate $\sigma _{k_1} \otimes \sigma _{k_2}(Y)$. Using (4.5) and (4.10) we find

$$\begin{aligned} {[\sigma _{k_1} \otimes \sigma _{k_2}(Y)f]}(x_1,x_2)= & {} -2k_1k_2 f(x_1,x_2)-2(x_1k_2-x_2k_1)\Big (\frac{\partial }{\partial x_1} - \frac{\partial }{\partial x_2} \Big ) f(x_1,x_2) \nonumber \\&+\, x_1x_2 \Big (\frac{\partial ^2}{\partial x_1^2} -2\frac{\partial ^2}{\partial x_1\partial x_2} + \frac{\partial ^2}{\partial x_2^2} \Big )f(x_1,x_2), \end{aligned}$$

which corresponds to the term with $(i,j) = (1,2)$ in (4.2). $\square$

Finally, application of Theorem 2.2 gives duality between the symmetric inclusion process SIP(*k*) and the Brownian energy process BEP(*k*).

#### Theorem 4.15

The operators $L^{\mathrm {SIP}}$ defined by (4.1) and $L^{\mathrm {BEP}}$ defined by (4.2) are in duality, with duality function

$$\begin{aligned} \prod _{j=1}^N L(n_j,x_j;k_j). \end{aligned}$$

### Bessel Functions and Self-Duality of BEP

The Bessel function of the first kind [1, Chapter 4] is defined by

$$\begin{aligned} J_\nu (x) = \frac{ (x/2)^\nu }{\Gamma (\nu +1)} \,_{0}F_{1}\!\left( \genfrac{}{}{0.0pt}{}{\text {--}}{\nu +1} \,;-\frac{x^2}{4} \right) . \end{aligned}$$

The function $J_\nu (xy)$ is an eigenfunction of a second-order differential operator;

$$\begin{aligned} T=-\frac{\partial ^2}{\partial x^2} - \frac{1}{x} \frac{\partial }{\partial x} + \frac{\nu ^2}{x^2} , \qquad TJ_\nu (xy) = y^2 J_\nu (xy). \end{aligned}$$

The Hankel transform is a unitary operator ${\mathcal {F}}_\nu : L^2([0,\infty ),x dx) \rightarrow L^2([0,\infty ),ydy)$ defined by

$$\begin{aligned} ({\mathcal {F}}_\nu f)(y) = \int _0^\infty f(x) J_\nu (xy) x\, dx, \qquad \nu >-1, \end{aligned}$$

for suitable functions *f*, and the inverse is given by ${\mathcal {F}}_\nu ^{-1} = {\mathcal {F}}_\nu$.

Let $k>0$. We consider the second-order differential operator $\sigma _k(F)$, see (4.10). Using the differential equation for the Bessel functions, we can find eigenfunctions of $\sigma _k(F)$ in terms of the Bessel functions

$$\begin{aligned} J(x,y;k) = e^{\frac{1}{2}(x+y)} (xy)^{-k+\frac{1}{2}} J_{2k-1}(\sqrt{xy}). \end{aligned}$$

We can also determine the actions of *H* and *E* on the eigenfunctions.

#### Lemma 4.16

The Bessel functions $J(x,y)=J(x,y;k)$ satisfy

$$\begin{aligned} \begin{aligned} {[\sigma _k(H)J(\cdot ,y)]}(x)&= -2y \frac{\partial }{\partial y}J(x,y) - (2k-y)J(x,y),\\ {[\sigma _k(E)J(\cdot ,y)]}(x)&= 2iy \frac{\partial ^2}{\partial y^2} J(x,y) + 2i(2k-y) \frac{\partial }{\partial y} J(x,y) -\frac{i}{2}(4k-y) \,J(x,y),\\ {[\sigma _k(F) J(\cdot ,y)]}(x)&= \frac{1}{2}iy\, J(x,y). \end{aligned} \end{aligned}$$

#### Proof

The action of *F* follows from the differential equation for the Bessel functions. We have

$$\begin{aligned} {[\sigma _k(E)J(\cdot ,y)]}(x)=-\frac{ix}{2} J(x,y), \end{aligned}$$

then using the self-duality of the Bessel functions, i.e. symmetry in *x* and *y*, we obtain the action of *E*. Finally, having the actions of *E* and *F*, we find the action of *H* from $H=[E,F]$. $\square$

Using the Hankel transform we can now define a unitary intertwiner with a kernel that has the desired properties.

#### Proposition 4.17

The operator $\Lambda :{\mathcal {P}} \rightarrow F([0,\infty ))$ defined by

$$\begin{aligned} (\Lambda f)(y) = \int _0^\infty f(x) J(x,y) w(x;k)\, dx, \end{aligned}$$

extends to a unitary operator $\Lambda :\mathrm H_k \rightarrow \mathrm H_k$ intertwining $\sigma _k$ with itself. Furthermore, the kernel satisfies

$$\begin{aligned} {[\sigma _k(X^\star ) J(\cdot ,y)]}(x) = [\sigma _k(X) J(x,\cdot )](y). \end{aligned}$$

#### Proof

Since the set of polynomials ${\mathcal {P}}$ is dense in $\mathrm H_k$, it is enough to define $\Lambda$ on ${\mathcal {P}}$. Unitarity of $\Lambda$ is essentially unitarity of the Hankel transform ${\mathcal {F}}_{2k-1}$. The intertwining property follows directly from Lemma 4.16. $\square$

Note that $\Lambda$ intertwines between $*$-representation with respect to the $*$-structure given by (4.9). Equivalently, $\Lambda$ intertwines $\sigma \circ \theta ^{-1}$ with itself, which is a $*$-representation with respect to the $\mathfrak {su}(1,1)$-$*$-structure.

Let $k \in {\mathbb {R}}_{>0}^N$, and consider the tensor product representation $\sigma _k$ defined by (4.11). Then from Proposition 4.17, Lemma 4.14 and Theorem 2.2 we obtain self-duality of the Brownian energy process BEP(*k*).

#### Theorem 4.18

The operator $L^{\mathrm {BEP}}$ given by (4.2) is self-dual, with duality function given by

$$\begin{aligned} \prod _{j=1}^N J(x_j,y_j;k_j). \end{aligned}$$

### More Duality Relations

The Meixner polynomials from Sect. 4.1 can be considered as overlap coefficients between eigenvectors of the elliptic Lie algebra element *H* and another elliptic element $X_{a(c)}$. There is a similar interpretation as overlap coefficients for the Laguerre polynomials (elliptic *H* - parabolic $X_1$) and Bessel functions (parabolic $X_1$ - parabolic $X_{-1}$). So far we did not consider overlap coefficients involving a hyperbolic Lie algebra element, because there does not seem to be an interpretation in this setting for the element *Y* from (4.5) as generator for a Markov process. However, the construction we used still works and leads to duality as operators between $L^{\mathrm {SIP}}$ or $L^{\mathrm {BEP}}$ and a difference operator $L^{\mathrm {hyp}}$ defined below, which may be of interest. We will give the main ingredients for duality between $L^{\mathrm {SIP}}$ and $L^{\mathrm {hyp}}$ in case $N=2$ using overlap coefficients between elliptic and hyperbolic bases, which can be given in terms of Meixner–Pollaczek polynomials, see also [9, 12].

The Meixner–Pollaczek polynomials [11, Section 9.7] are defined by

$$\begin{aligned} P_n^{(\lambda )}(x;\phi ) = e^{in\phi }\frac{ (2\lambda )_n}{n!} \,_{2}F_{1}\!\left( \genfrac{}{}{0.0pt}{}{-n,\lambda +ix}{2\lambda } \,;1-e^{-2i\phi } \right) . \end{aligned}$$

The orthogonality relations are

$$\begin{aligned} \frac{1}{2\pi }\int _{-\infty }^\infty P_m(x)P_n(x)\, e^{(2\phi -\pi )x} |\Gamma (\lambda +ix)|^2 \, dx \!=\! \delta _{mn} \frac{ \Gamma (n+2\lambda ) }{(2\sin \phi )^{2\lambda } \, n!}, \qquad \lambda >0,\ 0< \phi < \pi , \end{aligned}$$

and the Meixner–Pollaczek polynomials form an orthogonal basis for the corresponding weighted $L^2$-space. The three-term recurrence relations is

$$\begin{aligned} 2x \sin \phi \, P_n(x) = (n+1)P_{n+1}(x) - 2(n+\lambda ) \cos \phi \, P_n(x) + (n+2\lambda -1) P_{n-1}(x), \end{aligned}$$

and the difference equation is

$$\begin{aligned} 2(n\!+\!\lambda )\sin \phi \, P_n(x) \!=\! -ie^{i\phi } (\lambda \!-\!ix)\, P_n(x\!+\!i)\! +\! 2 x \cos \phi \, P_n(x) + ie^{-i\phi } (\lambda +ix) P_n(x-i). \end{aligned}$$

We also need the representation $\rho _k$ on $\mathrm {H}_k^\phi =L^2({\mathbb {R}}, w_k^\phi (x)dx)$, with weight function

$$\begin{aligned} w_k^\phi (x) = \frac{(2\sin \phi )^{2k} }{2\pi \Gamma (2k)} e^{-\pi x} |\Gamma (k+ix)|^2, \end{aligned}$$

given by

$$\begin{aligned} \begin{aligned} {[\rho _k(H) f]}(x)&= 2ix f(x),\\ {[\rho _k(E) f]}(x)&= (k-ix) f(x+i),\\ {[\rho _k(F) f]}(x)&=-(k+ix) f(x-i). \end{aligned} \end{aligned}$$

Now define an operator $L^{\mathrm {hyp}}$ on an appropriate dense subspace of $\mathrm {H}_{k_1}^{\phi } \otimes \mathrm {H}_{k_2}^\phi$ by

$$\begin{aligned} L^{\mathrm {hyp}} = \rho _{k_1} \otimes \rho _{k_2}(Y) + k_1k_2, \end{aligned}$$

where *Y* is given by (4.5), then we see that $L^{\mathrm {hyp}}$ is the difference operator given by

$$\begin{aligned} {[L^{\mathrm {hyp}} f]}(x_1,x_2)= & {} 2(k_1-ix_1)(k_2+ix_2) \Big ( f(x_1+i,x_2-i) - f(x_1,x_2) \Big ) \nonumber \\&+\, 2(k_1+ix_1)(k_2-ix_2) \Big ( f(x_1-i,x_2+i) - f(x_1,x_2)\Big ). \end{aligned}$$

In order to obtain a duality relation, we use the Lie algebra isomorphism $\theta _\phi : \mathfrak {sl}(2,{\mathbb {C}}) \rightarrow \mathfrak {sl}(2,{\mathbb {C}})$ defined by

$$\begin{aligned} \begin{aligned} \theta _\phi (H)&= \frac{i}{\sin \phi }\Big ( -\cos \phi H + E - F\Big ),\\ \theta _\phi (E)&= \frac{1}{2i\sin \phi }\Big (- H + e^{-i\phi } E - e^{i\phi }F\Big ),\\ \theta _\phi (F)&= \frac{1}{2i\sin \phi }\Big ( - H + e^{i\phi } E - e^{-i\phi }F\Big ). \end{aligned} \end{aligned}$$

Note that $\theta _\phi (H) = \frac{i}{\sin \phi }X_{\cos \phi }$, see (4.6), which is a hyperbolic Lie algebra element. The isomorphism does not preserve the $\mathfrak {su}(1,1)$-$*$-structure, but we have $\theta _\phi (X^*) = \theta _\phi (X)^\star$, see (4.9). Now consider the functions

$$\begin{aligned} P(n,x)=P(n,x;k,\phi ) = e^{x\phi }\frac{n!}{(2k)_n} P^{(k)}_n(x;\phi ). \end{aligned}$$

Using $H= \frac{-i}{\sin \phi } ( \cos \phi (\theta _\phi (H)) - \theta _\phi (E) - \theta _\phi (F))$, the three-term recurrence relation and the difference equation, one finds

$$\begin{aligned} \begin{aligned} {[\pi _k(\theta _\phi (H)) P(\cdot ,x)]}(n)&= 2ix\, P(n,x),\\ {[\pi _k(\theta _\phi (E)) P(\cdot ,x)]}(n)&= -(k-ix) P(n,x+i),\\ {[\pi _k(\theta _\phi (F)) P(\cdot ,x)]}(n)&= (k+ix) P(n,x-i). \end{aligned} \end{aligned}$$

so that

$$\begin{aligned} {[\pi _k(X^*) P(\cdot ,x)]}(n) = [\rho _k(\theta _\phi ^{-1}(X)) P(n,\cdot )](x). \end{aligned}$$

Then we can construct a unitary intertwiner between $\pi _k\circ \theta _\phi$ and $\rho _k$ with *P*(*x*, *n*) as a kernel, but we do not actually need the intertwiner, since the kernel is enough to state the duality result. Using $\theta _\phi (\Omega )=\Omega$ we obtain duality between the operators $L^{\mathrm {SIP}}$ and $L^{\mathrm {hyp}}$, with duality function given by the product

$$\begin{aligned} P(n_1,x_1;k_1,\phi ) P(n_2,x_2;k_2,\phi ). \end{aligned}$$

In a similar way we can find duality between $L^{\mathrm {BEP}}$ and $L^{\mathrm {hyp}}$ in terms of Laguerre functions, and also self-duality for $L^{\mathrm {hyp}}$ in terms of Meixner–Pollaczek functions.

## References

[1] Andrews GE, Askey R, Roy R (1999). Special Functions, Encycl. Math. Appl..

[2] Basu D, Wolf KB (1982). The unitary irreducible representations of $$\rm SL(2,{\mathbb{R}})$$ in all subgroup reductions. J. Math. Phys..

[3] Carinci G, Giardinà C, Giberti C, Redig F (2015). Dualities in population genetics: a fresh look with new dualities. Stoch. Process. Appl..

[4] Corwin, I.: Two ways to solve ASEP. In: Topics in Percolative and Disordered Systems. Springer Proc. Math. Stat., vol. 69, pp. , 1–13. Springer, New York (2014)

[5] Franceschini, C., Giardinà, C.: Stochastic duality and orthogonal polynomials. arXiv:1701.09115 [math.PR]

[6] Giardinà C, Kurchan J, Redig F (2007). Duality and exact correlations for a model of heat conduction. J. Math. Phys..

[7] Giardinà C, Kurchan J, Redig F, Vafayi K (2009). Duality and hidden symmetries in interacting particle systems. J. Stat. Phys..

[8] Groenevelt W, Koelink E (2002). Meixner functions and polynomials related to Lie algebra representations. J. Phys. A.

[9] Groenevelt, W., Koelink, E., Rosengren, H.: Continuous Hahn functions as Clebsch-Gordan coefficients. In: Theory and Applications of Special Functions. Dev. Math., vol. 13, pp. 221–284. Springer, New York (2005)

[10] Jansen S, Kurt N (2014). On the notion(s) of duality for Markov processes. Probab. Surv..

[11] Koekoek R, Lesky PA, Swarttouw R (2010). Hypergeometric Orthogonal Polynomials and Their q-Analogues.

[12] Koelink HT, Van der Jeugt J (1998). Convolutions for orthogonal polynomials from Lie and quantum algebra representations. SIAM J. Math. Anal..

[13] Kipnis C, Marchioro C, Presutti E (1982). Heat flow in an exactly solvable model. J. Stat. Phys..

[14] Koornwinder, T.H.: Group theoretic interpretations of Askey’s scheme of hypergeometric orthogonal polynomials. In: Orthogonal Polynomials and Their Applications (Segovia, 1986). Lecture Notes in Math., vol. 1329, pp. 46–72. Springer, Berlin (1988)

[15] Liggett, T.M.: Interacting Particle Systems. Classics in Mathematics. Springer, Berlin (2005) (**Reprint of the 1985 original**)

[16] Redig, F., Sau, F.: Duality functions and stationary product measures. arXiv:1702.07237 [math.PR] 10.1007/s10955-018-2090-1 PMC6434980 30996474

[17] Spitzer F (1970). Interaction of Markov processes. Adv. Math..

[18] Spohn H (1983). Long range correlations for stochastic lattice gases in a nonequilibrium steady state. J. Phys. A.

[19] Sturm, A., Swart, J.M., Völlering, F.: The algebraic approach to duality: an introduction. arXiv:1802.07150 [math.PR]

